# Characterization and performance of castor bean lineages and parents at the UFRB germplasm bank

**DOI:** 10.1371/journal.pone.0209335

**Published:** 2019-01-07

**Authors:** Adielle Rodrigues da Silva, Simone Alves Silva, Laurenice Araujo dos Santos, Deoclides Ricardo de Souza, Gilmara de Melo Araujo, Jorge Luiz Loyola Dantas, Elton da Silva Leite, Ana Cristina Vello Loyola Dantas

**Affiliations:** 1 Centro de Ciências Agrárias, Ambientais e Biológicas, Universidade Federal do Recôncavo da Bahia (UFRB), Cruz das Almas, BA, Brasil; 2 Embrapa Mandioca e Fruticultura, Cruz das Almas, BA, Brasil; USDA-ARS, UNITED STATES

## Abstract

The objective of this work was to characterize 203 lineages and five parents of *Ricinus communis* L. from the germplasm bank at the Federal University of Recôncavo da Bahia (UFRB), which was established by the Genetic Improvement and Biotechnology Program (NBIO) at the Center for Agrarian, Environmental and Biological Sciences. The study used 35 morpho-agronomic descriptors, proposed by the Ministry of Agriculture, Livestock and Supply, and 12 quantitative descriptors suggested by NBIO. The experiment was conducted in a randomized block design, composed of four blocks, in the experimental field at UFRB in 2014. The frequency and entropy level of the qualitative descriptors were estimated with the Renyi procedure, and an analysis of variance was used for the quantitative descriptors. The analyses were made with the statistical program R. Of the qualitative morpho-agronomic descriptors evaluated, 22.86% had a high level of entropy (above 1.0), and all 12 quantitative descriptors showed significant differences. This indicates genetic variability in the germplasm bank and a satisfactory performance for most of the descriptors evaluated, as well as the possibility of direct and indirect use of the lineages and parents in genetic improvement programs of the species.

## Introduction

Castor bean (*Ricinus communis* L.) is a socially and economically important plant because the oil extracted from its seeds has unique chemical properties that make it widely useful, for example, to the biomedical and chemical industries, and to produce biodiesel [1].

Morphological descriptors of this species are highly variable, such as the color of the leaves, stems, stigma and fruits, and presence or absence of wax on the stem and fruits [2, 3]. Based on this, it is important to characterize the castor bean germplasm in banks because understanding the different genetic constituents of the germplasm will aid the immediate development of new cultivars and future genetic improvement programs.

One form of characterization is morpho-agronomic, which consist of describing morphological characters of a species, has the advantage of being inexpensive compared to other characterization types, is based on the phenotype, and results in a greater amount of information. Various works have used this technique to characterize many species, for example, in studies of castor bean by [4, 5, 6]. In these works, variability was verified for most of the descriptors evaluated, proving that the species presents highly variable morpho-agronomic descriptors. Thus, qualitative and quantitative descriptors contribute to morpho-agronomic characterization, mainly when evaluating performance among lineages.

In this sense, due to the great variability of the germplasm, both morphological and demographic characterizations are crucial for the effective management of the collections of germplasm to facilitate the use of accessions in future breeding programs [7].

The morphoagronomic characterization can be evaluated by several methods, including the entropy level by the Renyi coefficient, which is a measure of the frequency of distribution of the lineages in each of the phenotypic classes of the qualitative descriptor evaluated, indicating the presence or absence of variability. This methodology applied by [7] to morphological descriptors and found in the Embrapa Cerrados cassava germplasm Bank a high level of entropy for the descriptors external stem color, petiole color, central lobe shape and color of the apical leaf. Some studies have applied the methodology of frequency analysis of the phenotype classes in castor bean, such as that of [6], showing variability in the characters coloring, racemic shape and type of branching, fruit density, dehiscence and the presence of spines. Accessions evaluated are sources of germplasm for breeding programs. Already [8] characterized 40 Kashmiri apricot genotypes for 28 morphological characteristics and found a high level of variability. Characteristics of the fruit, such as size, shape, fruit volume, skin color and meat, which determine the quality and commercialization of apricots.

This work evaluated and morpho-agronomically characterized 203 lineages and five parents from the castor bean germplasm bank (BAG) of the Genetic Improvement and Biotechnology Program (NBIO), which is part of the Center for Agrarian, Environmental and Biological Sciences (CCAAB) at the Federal University of Recôncavo da Bahia (UFRB). The study used morpho-agronomic descriptors proposed by the Ministry of Agriculture, Livestock and Supply (Mapa) and NBIO.

## Material and methods

### Characterization of the area

The study was conducted from 2014 to 2015 in the experimental area of CCAAB at UFRB, on the campus of Cruz das Almas—BA. The municipality (12°40'39" S, 39°40'23" W) is at 220 m elevation and has an average temperature of 24.5°C, relative humidity of 82%, and annual rainfall of 1,197 mm. According to the Köppen classification system, the climate in the region is a transition zone between Am (monsoon climate) and Aw (clima tropical com estação seca de Inverno) (type C1), and is dry and subhumid. The soil in the experimental field is classified as a dystrophic yellow latosol A—moderate sandy clay texture [9].

### Plant material

Castor bean lineages maintained in the BAG of UFRB/CCAAB/NBIO were evaluated using phenotypic characters (morphological and agronomic descriptors). The study population was an advanced generation with a high level of homogeneity developed by the castor bean Genetic Improvement Program of NBIO at UFRB. It was obtained by self-fertilization using single seed descent (SSD), where the segregating F_2_ population was acquired by self-fertilization of the F_1_ population that resulted from the hybridization of diverse parental accessions (BRS 149 Nordestina, BRS 188 Paraguaçu, EBDA MPA-17, Mirante 10, and Sipeal 28) [10].

There were 208 treatments, comprising 203 lineages and five parents. The experiment was in a randomized block design, with four repetitions, 3 m of space between rows, 1 m between plants, and 2 meters between blocks.

The planting area was fertilized with 20 kg ha^-1^ of N, 80 kg ha^-1^ of P, and 40 kg ha^-1^ of K. In May 2014, three seeds of each lineage and each parent were planted in pits. Twenty-five days after germination the seedlings were thinned to 1 plant per pit.

During the experiment, spontaneous plants were controlled with manual weeding and two cover fertilizers were applied, urea and potassium chloride in the proportion of 2:1 (20 g and 10 g per plant), respectively. Gray mold (*Amphobotrys ricini*) during flowering was controlled with fungicides.

### Characterization morpho-agronomic at the germplasm bank

Each lineage was morphologically characterized while it developed in the field, which was based on phenotypic classes of descriptors presented in the “Formulário de Instruções para Execução dos Ensaios de Distinguibilidade, Homogeneidade e Estabilidade de Cultivares de Mamona (Ricinus communis L.)” published by Mapa [11], images in “Documento 192” published by Embrapa [12], and descriptors suggested by NBIO (Table 1).

**Table 1:** Descriptors of castor bean proposed by Mapa [11] and NBIO.

**Descriptors proposed by Mapa [11]**			
**Descriptors**	**Evaluation**		**Phenotypic classes**
Up to 10 days after emergence.			
1. Anthocyanin Pigmentation (AP).	Observe visually for the presence or absence of anthocyanin pigmentation in the hypocotyl. Fig 1(A), 1(B) and 1(C).		1. Absent. 2. Present.
Full flowering of the primary raceme.			
2. Insertion of the Primary Raceme (IPR).	Measurement from the soil to the first point of the primary raceme.		1. Low (< 50 cm). 2. Middle (51 to 100 cm). 3. High (> 100 cm).
3. Stem Diameter (SD).	The middle third of the stem, using a digital caliper.		1. Thin (< 3 cm). 2. Medium (3 to 6 cm). 3. Thick (> 5 cm).
4. Average Internode Length (AIL).	Obtained the from NIC/IRP.		1. Short (< 2 cm). 2. Medium (3 to 5 cm). 3. Long (> 5 cm).
5. Number of Stem Internodes (NSI).	Count the number of scars on the stem.		1. Low (up to 15). 2. Medium (16 to 18). 3. High (> 19).
6. Stem Wax (SW).	Record the presence or absence of wax on the stem.		1. Absent. 2. Present.
7. Stem Coloration (SC).	Based on images in Document 192 by Embrapa [9] and the phenotypic classes of Mapa [11]. Fig 1(D), 1(E), 1(F), 1(G), 1(H) and 1(I).		1. Light green. 2. Medium green. 3. Dark green. 4. Pinkish green. 5. Pinkish. 6. Red. 7. Reddish brown. 8. Purple.
8. Adaxial Leaf Surface (ALS).	The curvature of the adaxial surface of the leaf blade.		1. Flat. 2. Slightly concave. 3. Concave.
9. Pigmentation of Primary Veins (PPV).	Coloration of the veins on the abaxial surface of mature leaves. Fig 2(A) e 2(B).		1. Greenish. 2. Reddish.
10. Wax on Adaxial Blade Surface (WABS).	Observe the presence or absence of wax on the adaxial surface of mature leaves.		1. Absent. 2. Present.
11. Coloration of the Adaxial Blade Surface (CABS).	Color of the adaxial blade surface of mature leaves below primary raceme. Fig 2(C), 2(D) and 2(E).		1. Light green. 2. Medium green. 3. Dark green. 4. Pink. 5. Reddish green. 6. Red. 7. Purple.
12. Staminate Flowers on the Raceme (SFR).	Verify the presence or absence of staminate flowers.		1. Absent. 2. Present.
13. Location of Staminate Flowers (LSF).	Observe if the staminate flowers are above, below of interspersed with the pistillate flowers on the primary raceme.		1. Predominantly on the lower part of the raceme. 2. Interspersed with the pistillate flowers.
14. Stigma Coloration (SC).	Observe the color of the stigmas, before pollination, on the primary raceme. Fig 2(F), 2(G), 2(H), 2(I), 2(J) and 2(K).		1. Yellowish. 2. Greenish. 3. Orangish. 4. Reddish. 5. Purplish.
From the time of emergence to the initial pistillate flowering on the primary raceme.			
15. Flowering (FLO).	Subtraction of the date of germination from the date of flowering.		1. Precocious (up to 30 days). 2. Medium (31 to 60 days). 3. Late (Over 60 days).
Full flowering of the last commercial inflorescence.			
16. Plant Stature (PS).	Measurement from the soil to the highest branch of the plant.		1. Very short (< 100 cm). 2. Short (101 to 150 cm). 3. Medium (151 to 200 cm). 4. Tall (201 to 250 cm). 5. Very tall (> 250 cm).
17. Plant Architecture (PA).	Photograph the plant for analysis. Fig 3(A), 3(B) and 3(C).		1. Erect. 2. Semierect. 3. Open.
18. Number of Racemes Harvested (NRH).	Count number of racemes emitted by each plant.		1. Low (to 3). 2. Medium (4 to 7). 3. High (> 7).
19. Length of Primary Raceme (LPR).	Measure with a ruler from the apex of the 1° raceme to the scar of the first node.		1. Short (< 31 cm). 2. Medium (31 to50 cm). 3. Long (> 51 cm).
Immature fruits on the primary raceme.			
20. Density of the Raceme (DR).	Evaluation of the second raceme, since the primary raceme was self-fertilized and this could compact the raceme and interfere with the result. Fig 3(D), 3(E) and 3(F).		1. Sparse. 2. Intermediary. 3. Compact.
21. Raceme Shape (RS).			1. Globose. 2. Cylindrical. 3. Conical.
22. Fruit Wax (FW).	Evaluated visually.		1. Absent. 2. Present.
23. Fruit Coloration (FC).	Based on images in Document 192 by Embrapa [9] and the phenotypic classes of Mapa [11]. Fig 3(G), 3(H) and 3(I).		1. Yellowish. 2. Light green. 3. Medium green. 4. Dark green. 5. Pinkish green. 6. Pink. 7. Red. 8. Purple.
24. Presence of Prickles on the Fruits (PPF).	Visually evaluated.		1. Absent. 2. Present.
25. Density of the Prickles on the Fruits (DPF).			1. Low. 2. Medium. 3. High.
26. Coloration of the Fruit Prickles (CFP).	Based on images in Document 192 by Embrapa [9] and the phenotypic classes of Mapa [11].		1. Yellowish. 2. Light green. 3. Medium green. 4. Dark green. 5. Pinkish green. 6. Pink. 7. Red. 8. Purple.
Mature fruits or racemes.			
27. Fruit Dehiscence (FD).	Based on quantity of open fruits.		1. Dehiscent. 2. Semidehiscent. 3. Indehiscent.
Seeds harvested from mature fruits			
28. Presence of Secondary Coloration (PSC).	Visually evaluated.		1. Absent. 2. Present.
29. Main Seed Coloration (MSC).	Corresponds to the predominant color.		1. White. 2. Yellowish. 3. Reddish. 4. Light brown. 5. Medium brown. 6. Dark brown. 7. Reddish brown. 8. Grayish. 9. Black.
30. Secondary Coloration of Seeds (SCS).	Based on images in Document 192 by Embrapa [9] and the phenotypic classes of Mapa [11].		
31. Type of Secondary Coloration (TSC).	Classified as painted when there are small marks, cracked when there are elongated designs, and scored when there are irregularly distributed marks. Fig 3(J), 3(K), 3(L), 3(M), 3(N) and 3(O).		1. Painted. 2. Cracked. 3. Scored.
32. Seed Shape (SS).	Visually evaluated.		1. Round. 2. Ellipsoid.
33. Caruncle Protuberance (PDC).	Visually evaluated.		1. Slight. 2. Prominent.
34. Weight of 100 seeds at 9% moisture content (W100).	Moisture content of seeds based on RAS [31] using a dryer at 105°C and the rule of three to determine the weight of 100 seeds at 9% moisture content.		1. Low (< 40 g). 2. Medium (41 to 55 g). 3. High (> 55 g).
35. Seed Yield per Fruit (SYF).	Percentage of weight of the seeds divided by the weight of the fruits.		1. Low (< 60%). 2. Medium (61 to 80%). 3. High (> 80%).
**Descriptors Suggested by NBIO (2014)**			
**Descriptors**		**Evaluation**	
36. Number of Fruits per Raceme (NFR).		Average count of the number of fruits on the first four racemes.	
37. Number of Seeds on the Primary Raceme (NSPR).		Count of the number of seeds on the primary raceme.	
38. Number of Seeds per Raceme (NSR).		Average count of the number of seeds from the first four racemes.	
39. Seed Weight per Raceme (SWR).		Average weight of the seeds of the first four racemes, using an analytical balance	
40. Seed Weight per Plant (SWP).		Sum of the weight of the seeds from the first four racemes, using an analytical balance.	
41. Number of Fruits per Plant (NFP).		Count of the number of fruits on the first four racemes.	
42. Number of Seeds per Plant (NSP).		Count of the number of seeds from the first four racemes.	
43. Weight of the Racemes per Plant (WRP).		Weight of the first four racemes, using an analytical balance.	
44. Weight of the Fruits per Raceme (WFR).		Average weight of the fruits from the first four racemes, using an analytical balance.	
45. Productivity (PROD).		Calculated estimate of kg ha^-1^ for each plant.	
46. Raceme Length (RL).		Average length of the first four racemes.	
47. Effective Length of the Raceme (ELR).		Measurement from the apex of the raceme to the last peduncle.	

**Fig 1:**
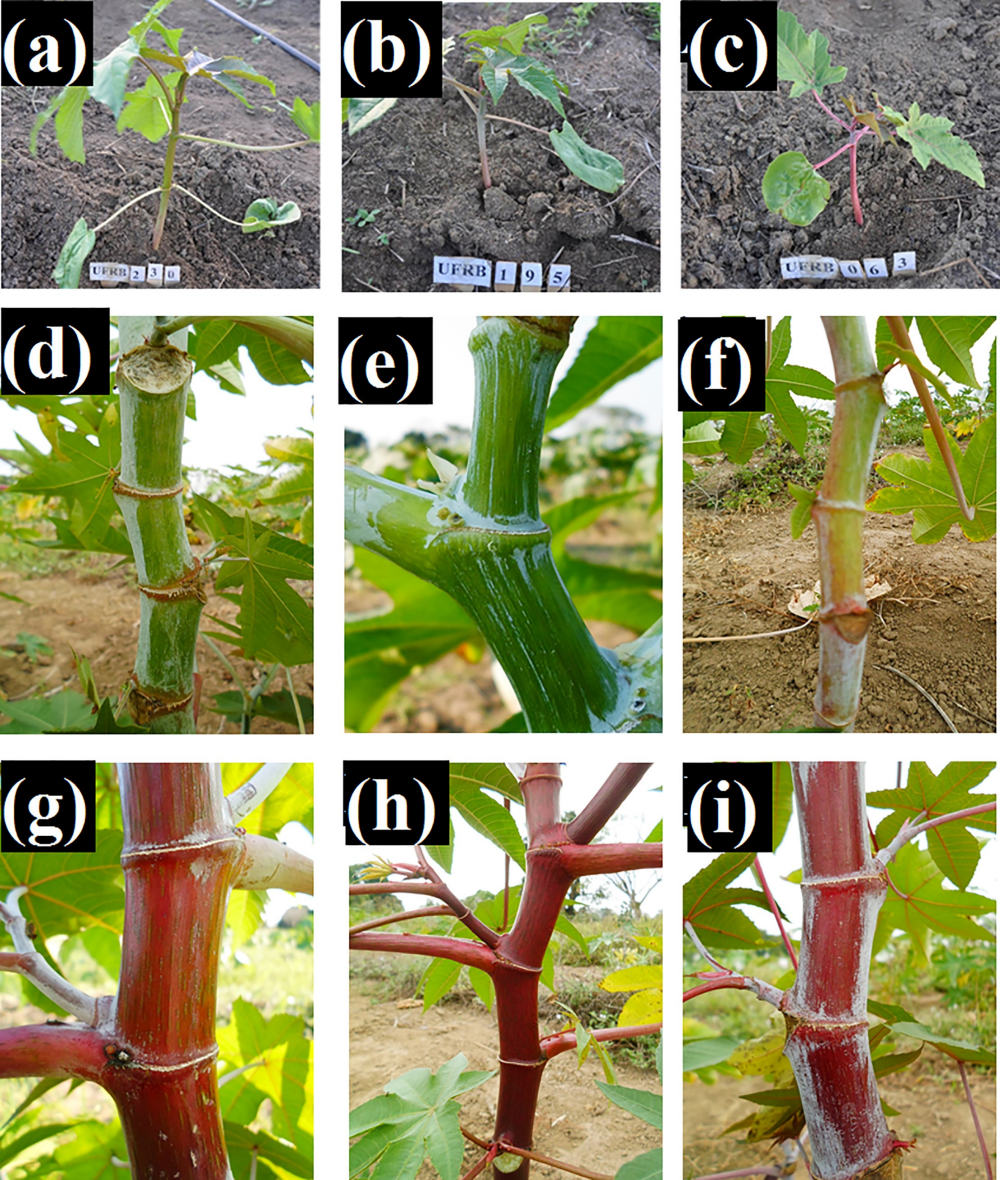
Morphoagronomic descriptors evaluated in 203 lineages and five parental castor beans from the germplasm bank of the UFRB / CCAAB / NBIO. Cruz das Almas—BA, 2014. (a), (b) and (c) Anthocyanin pigmentation; and (d), (e), (f), (g), (h) and (i) Stem coloring. **Source:** Archive NBIO/ ARAÚJO & SILVA, 2014–2015.

**Fig 2:**
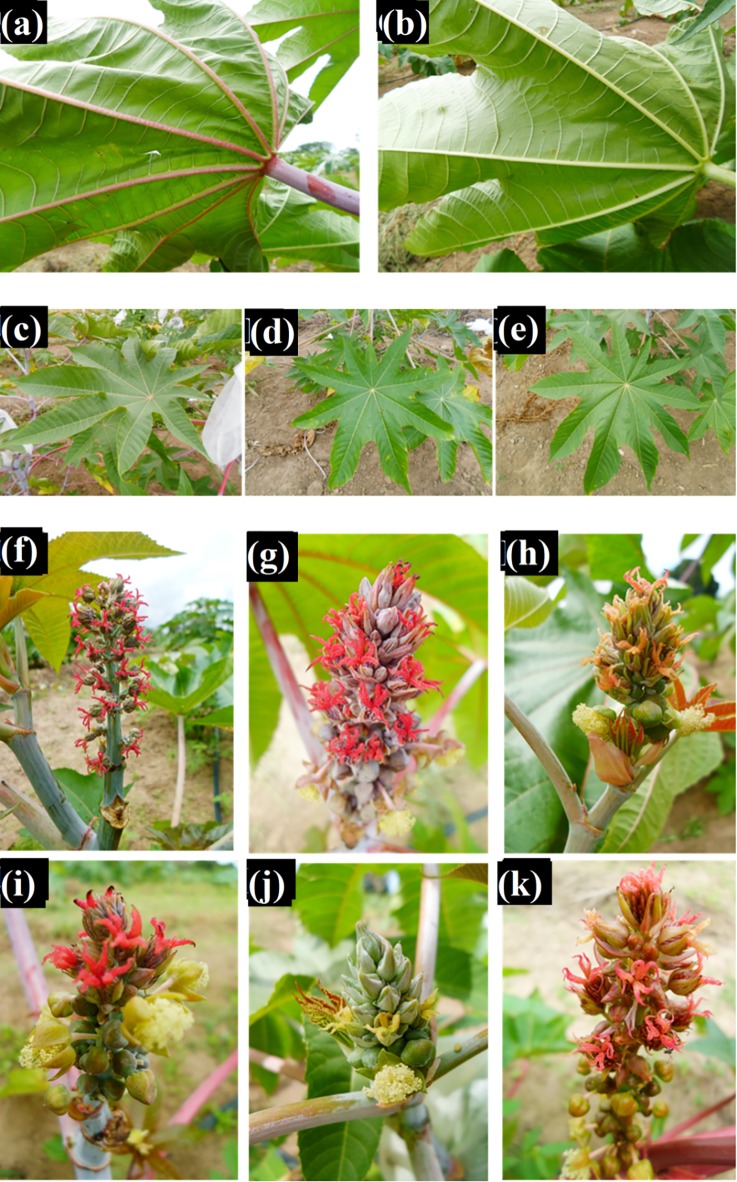
Morphoagronomal descriptors evaluated in 203 lineages and five parental castor bean from the germplasm bank of the UFRB / CCAAB / NBIO. Cruz das Almas—BA, 2014. (a) and (b) Pigmentation of the main vein; (c), (d) and (e) Coloring of the upper limbus phase; and (f), (g), (h), (i), (j) and (k) staining of the stigma. **Source:** Archive NBIO/ ARAÚJO & SILVA, 2014–2015.

**Fig 3:**
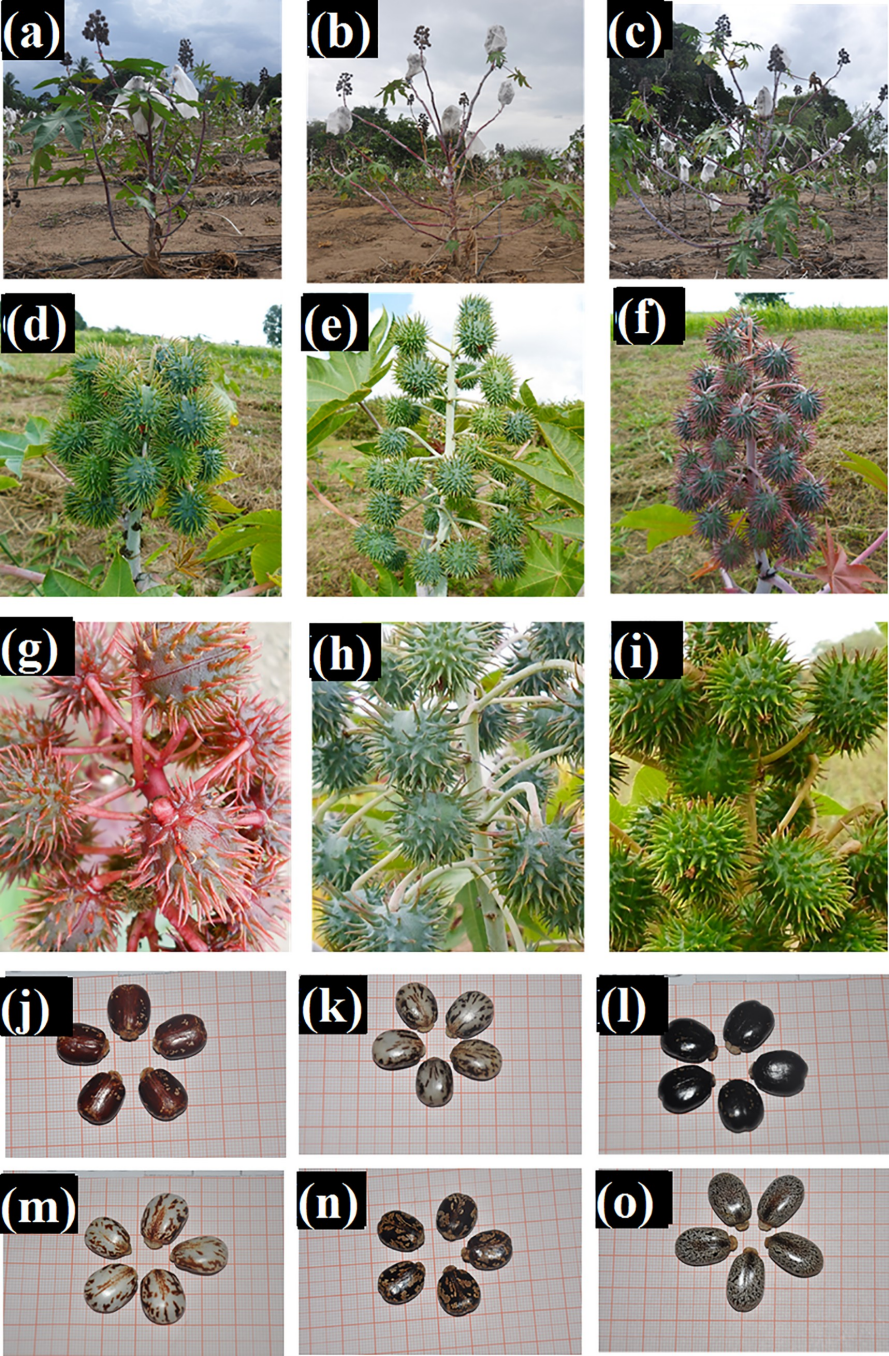
Morphoagronomal descriptors evaluated in 203 strains and five parental castor bean samples from the germplasm bank of the UFRB / CCAAB / NBIO. Cruz das Almas—BA, 2014. (a), (b) and (c) Architecture of the plant; (d), (e) and (f) Form of the racemic; (g), (h) and (i) Fruit color; and (j), (k), (l), (m), (n) and (o) seed coloration. **Source:** Archive NBIO/ ARAÚJO & SILVA, 2014–2015.

For the descriptors with distinct classes that were easily visualized, illustrative records were made by taking photographs. To take the best images, photographs were taken at the end of day when it was cloudy, so there was no interference from sunlight, and semiprofessional equipment was used (digital camera with 18–105 mm lens).

For the qualitative descriptors, the percentage frequencies of each category and the level of entropy of the characters (H) were calculated using the Renyi coefficient

$$H=-\sum_{i-l}^{s}pi\;\ln pi$$

The entropy is a measurement of the frequency of the distribution of (n) linages P = (p 1, p 2 … p s), where p 1 = f i/n and (p 1 + p 2 + … + p s = 1), as long as (n = f 1 + f 2 + … + f s), and f 1, f 2, … f n are the counts of each of the classes (s) of the descriptor considered [8]. For the quantitative descriptors, an analysis of variance was conducted to verify the level of variability. The analysis was made with the statistical program R [13].

## Results and discussion

According to [8], the entropy level (H) is related to the number of phenotypic classes and the ratio of these classes. The more classes there are and the more balanced the ratio between the frequencies, the higher the H. Thus, for a descriptor with two classes the highest H will occur when each class includes 50% of the accessions.

Table 2 lists the entropy level and frequency of the 35 qualitative descriptors evaluated. Of these, only anthocyanin pigmentation, wax on adaxial blade surface, presence of prickles on the fruits, presence of secondary coloration on the seed, and location of the staminate flowers on the raceme had one phenotypic class. Anthocyanin pigmentation was present in 100% of the population; however, visually some plants were more intense than others. [14] in a work about anthocyanin concentration in *Pseudobombax marginatum* using spectrophotometry, noted that low concentrations of anthocyanins could result in the absence of strong coloration. In turn, [15] explains that regardless of anthocyanin intensity, the stem staining inheritance is of a monogenic nature in that green staining is controlled by recessive alleles over dominant red staining. [16] The presence of this character has been related to resistance to *Fusarium* and some pests.

**Table 2:** Descriptors, phenotypic classes, frequencies, and entropy level evaluated for 203 lineages and five parents of castor bean from the germplasm bank at UFRB/NBIO. Cruz das Almas-BA, 2014–2015.

Descriptor	Classes	Frequency (%)	Entropy Level (H)
1. Anthocyanin Pigmentation.	1. Absent.	0,00	0,00
	2. Present.	100,00	
2. Insertion of the Primary Raceme.	1. Low.	24,04	0,60
	2. Middle.	75,00	
	3. High.	0,96	
3. Stem Diameter.	1. Thin.	96,15	0,16
	2. Medium.	3,85	
	3. Thick.	0,00	
4. Average Internode Length.	1. Short.	3,37	0,29
	2. Medium.	93,27	
	3. Long.	3,37	
5. Number of Stem Internodes.	1. Low.	29,81	1,07
	2. Medium.	44,23	
	3. High.	25,96	
6. Stem Wax.	1. Absent.	12,02	0,37
	2. Present.	87,98	
7. Stem Coloration.	1. Light green.	30,77	1,70
	2. Medium green.	13,46	
	3. Dark green.	1,92	
	4. Pinkish green.	10,10	
	5. Pinkish.	2,40	
	6. Red.	0,96	
	7. Reddish brown.	13,94	
	8. Purple.	26,44	
8. Adaxial Leaf Surface.	1. Flat.	14,90	1,01
	2. Slightly concaved.	38,46	
	3. Concaved.	46,63	
9. Pigmentation of Primary Veins.	1. Greenish.	54,33	0,69
	2. Reddish.	45,67	
10. Wax on Adaxial Blade Surface.	1. Absent.	100,00	0,00
	2. Present.	0,00	
11. Coloration of the Adaxial Blade Surface.	1. Light green.	0,48	0,64
	2. Medium green.	30,29	
	3. Dark green.	69,23	
12. Staminate Flowers on the Raceme.	1. Absent.	1,44	0,08
	2. Present.	98,56	
13. Location of Staminate Flowers.	1. Predominantly on the lower part of the raceme.	0,00	0,00
	2. Interspersed with the pistillate flowers.	100,00	
14. Stigma Coloration.	1. Yellowish.	5,77	1,11
	2. Greenish.	0,00	
	3. Orangish.	39,90	
	4. Reddish.	45,19	
	5. Purplish.	9,13	
15. Flowering.	1. Precocious.	0,00	0,18
	2. Medium.	6,73	
	3. Late.	93,27	
16. Density of the Raceme.	1. Sparse.	22,12	0,78
	2. Intermediary.	70,19	
	3. Compact.	7,69	

According to [8], in a work that morpho-angronomically characterized the germplasm of cassava, the elevated entropy of some descriptors used was due to the absence of agronomic interest in them. These descriptors where never used as selection criteria, either consciously in genetic breeding programs or unconsciously at the beginning of domestication of the species, and, for this reason, they maintain a high variability of classes.

Based on this, among the 35 qualitative descriptors evaluated, 22.86% exhibited diverse phenotypic classes that contributed to an elevated H: stigma coloration (1.11), with four phenotypic classes; coloration of the fruit prickles (1.69), with seven classes; main seed color (1.67), with nine classes; secondary seed coloration (1.22), with seven classes; and stem coloration (1.70), with eight classes, where the highest frequency was the light green class (30.77%) and the lowest was the red class (0.96%).

According to [15], red is controlled separately by a single dominant gene. [6] also found variability in stem color of castor bean, where 66.66% of the accessions evaluated had pink stems. Probably, the variability of classes for this descriptor is due a lack of preference for a specific color by the people who domesticated the plant. However, in the present work the elevated H for adaxial leaf surface (1.01), plant architecture (1.07), and number of stem internodes (NSI = 1.07) is due to the balanced frequencies among the classes of these descriptors. In relation to NSI, [17] commented that castor bean plants under conditions of excess water have more internodes.

It is important to characterize the germplasm in field conditions to verify its performance in normal conditions, [18] evaluating 100 accessions of sorghum sorghum verified a significant contribution of morphological and agroindustrial characteristics related to the production of sugar and biomass, being 53.1% for the analysis of phenotypic diversity.

These results indicate that the lineages of castor bean and the parents maintained in the BAG of UFRB/CCAAB/NBIO are genetically variable for the descriptors related to color, adaxial leaf surface, and number of stem internodes, and that this population has a broad genetic base for these descriptors. Thus, the variability detected is a desired result since it is important to maintain the variability of the species for its use in genetic improvement programs.

Among the qualitative descriptors evaluated, some exhibited a low H; stem diameter (0.16) is explained because it has only two phenotypic classes and an unequal distribution between them, where 96.15% of the population had thin stems. [19] in a study of populations of 25, 40, 55 and 70 thousand plants per hectare, verified that an increase in a population of castor bean plants of small size in an area reduces stem diameter, indicating that this character changes based on the environment, is adaptive, and should be evaluated in each specific environment. For mechanized harvesting, short plants with thin stems are recommended because thick stems make the process difficult. Thus, the evaluated lineages and the parents in BAG satisfy this characteristic of interest. Average H was calculated for the descriptor insertion of the primary raceme (IRP = 0.60), and the middle class had the highest frequency (75.00% of BAG.). This contributed to most lineages (93.27%) having long average internode lengths (0.29), because this descriptor is calculated by IPR/NSI. [20] explains that the insertion of the primary raceme corresponds to the initial reproductive phase, in that the primary stem grows until the primary raceme is emitted, and this phase is heavily influenced by environmental factors, such as photoperiod and daily temperature. This finding was verified by [17], where the insertion of the inflorescence was low and there was a low number of internodes on the stems for some castor bean plants under dry conditions. Other descriptors also had low entropy due to the low number of phenotypic classes and/or the unbalanced frequency among the classes, such as stem wax (0.37) where 87.98% of the castor bean plants had wax. According to [15], this character helps the plant tolerate cold and drought.

It is important to emphasize that descriptors of agronomic interest, for example, flowering (0,18), fruit dehiscence (0,73) and plant stature (0,75), also exhibited low to medium H, which is due to the use of these descriptors in the selection of superior lineages. Although they present medium and low entropy levels, these descriptors are important because they are correlated with other descriptors that help discriminate the accessions. [21] noted that the presence of the gene that determines the dwarf size of castor bean affects various distinct characteristics, such as curvature of the leaf blade, shorter internodes, undulating nodes, and little branching.

Table 2 shows that 93.27% of the castor bean plants from BAG flower late. For this character, it is ideal if plants can adjust flowering time based on environmental cultivation conditions so that lineages and parents can adapt to periods of drought. This reduces production costs, which is especially important to farmers with limited financial and technological resources. For the descriptor fruit dehiscence, 74.52% of the BAG accessions are semidehiscent. In relation to plant stature, most lineages are short (73.08%). [20] notes that a short plant is one of the goals of improvement programs because it exhibits less growth and is denser, making mechanized harvesting easier. [22] verified that an increase in the number plants in an area increases the stature of the plants due to the competition for light. The population of plants can also influence other descriptors, as observed by [19], where an increase in the number of short caster bean plants reduced the number of commercial racemes.

All 12 quantitative descriptors evaluated had significant differences based on the F test (Table 3), which is evidence of variability. According to [23], quantitative descriptors are polygenic and the number of phenotypic classes increases proportionally to the number of genes, reducing the differences between classes so there is a continuous distribution.

**Table 3:** Summary of the analysis of variance of 12 quantitative descriptors evaluated for 203 lineages and five parents of castor bean from the germplasm bank at UFRB/NBIO. Cruz das Almas-BA, 2014–2015.

Descriptors	Mean Square	F	Average	Minimum	Maximum	CV (%)
RL	31,46	1,51**	18,46	4,83	35,75	24,70
ELR	24,61	1,80**	11,20	1,83	28,07	32,95
WRP	1084,39	1,37**	69,87	5,66	180,63	40,31
NFR	116,36	1,46**	23,65	2,00	98,00	37,70
NFP	2013,80	1,43**	82,06	3,00	295,00	45,67
WFR	905,60	1,33**	64,98	5,18	168,21	40,12
SWR	369,59	1,39**	39,83	3,30	107,67	40,88
SWP	6399,44	1,30*	137,30	3,30	430,66	51,04
NSPR	2583,08	1,98**	67,97	0,00	233,00	53,07
NSR	962,70	1,49**	71,54	8,00	182,00	35,53
NSP	17284,96	1,43**	244,77	8,00	689,00	44,88
PROD	71104,67	1,30*	457,70	11,00	1436,00	51,04

*, ** probability of significance (p>0.05) and (p<0.01), respectively.

RL–Raceme length; ELR—Effective Length of the Raceme; WRP—Weight of the Racemes per Plant; NFR—Number of Fruits per Raceme; NFP—Number of Fruits per Plant; WFR–Weight of the Fruits per Raceme; SWR—Seed Weight per Raceme; SWP—Seed Weight per Plant; NSPR–Number of Seeds on the Primary Raceme; NSR—Number of Seeds per Raceme; NSP—Number of Seeds per Plant; and PROD–Productivity.

The experimental quality evaluated using the coefficient of variation (CV%) had limits of 24.70% and 53.07%. These values correspond to the length of the raceme and number of seeds of the primary raceme, respectively. The productivity descriptor had a value of 51.04%. These values agree with the quantitative and polygenic nature of these descriptors, which are strongly influenced by the environment [24]. CV% values for raceme length and productivity have been recorded by several authors: 13.42% and 32.84% [25]; 9.32% and 31.36% [4]; and 19.37% and 31.64% [5], respectively. [26] obtained a higher CV% value for productivity (54.88%).

The interval of variation obtained for the agronomic descriptors number of seeds per raceme (8 to 182 units) and seed weight per raceme (3.30 to 107.67 g) was very close to what has been observed in other works involving a segregated F_2_ generation from germplasm from BAG, such as [27] that found 35 to 163 units for seed number and 22.6 to 256 g for seed weight. [28] in an advanced population (F_3_:F_4_), obtained 73 to 157 units for seed number and 48.92 to 117.01 g for seed weight.

To date, 1,436.00 kg ha^-1 I^ is the highest productivity obtained for BAG germplasm. This is higher than the national average reported by the Instituto Brasileiro de Geografia e Estatística [29], which was 737 kg ha^-1^ in August 2015. [4] and [25] evaluated cultivars of castor bean in Recôncavo Baiano and observed that Sipeal 28 was the most productive (1,347 kg ha^-1^). [30] evaluated cultivars of castor bean in the region of Londrina (PR) and verified that the most productive cultivar was the variety IAC Guarani (911 kg ha^-1^).

## Conclusions

Variation in the descriptors was expected because in using a population obtained from SSD (Single Seed Descent) no selection occurred until a high level of homozygosity was reached. The variability encountered for the descriptors is important as a guide to help direct improvement programs of the species.

The descriptors related to color of the stigma, stem, prickles, and seeds exhibited elevated genetic variability, which is probably because these characteristics were not important during the domestication process.

The satisfactory results for the descriptors of agronomic interest suggest that selection within the evaluated population would be efficient because the advanced generation presented a high degree homozygosity and higher proportion of favorable alleles, which makes selection based on the phenotype more reliable.

## Supporting information

S1 Table Classificação de lineages and parents of castor bean from the germplasm bank at UFRB/NBIO for eight characteristics of agronomic interest. Cruz das Almas-BA, 2014–2015. Lines of castor bean from the germplasm bank of UFRB / CCAAB / NBIO, numbered from 1 to 265; and parental PAR (BRS 188 Paraguaçu), NOR (BRS 149 Northeast), MIR (Mirante 10), MPA (EBDA MPA-17) and SIP (Sipeal 28).
(DOCX)
