# Supplementary material for: Characterization and performance of castor bean lineages and parents at the UFRB germplasm bank
Source: PLoS One. 2019 Jan 7;14(1):e0209335. doi: 10.1371/journal.pone.0209335 (PMC6322771; doi:10.1371/journal.pone.0209335)
Supplement: S1 Table — Cruz das Almas-BA, 2014–2015. Lines of castor bean from the germplasm bank of UFRB / CCAAB / NBIO, numbered from 1 to 265; and parental PAR (BRS 188 Paraguaçu), NOR (BRS 149 Northeast), MIR (Mirante 10), MPA (EBDA MPA-17) and SIP (Sipeal 28). (DOCX) [file pone.0209335.s001.docx]

S1 Table:.Classificação de lineages and parents of castor bean from the germplasm bank at UFRB/NBIO for eight characteristics of agronomic interest. Cruz das Almas-BA, 2014-2015.

| **Descriptor** | **Lineages and five parents of castor bean** |
| --- | --- |
| Insertion of the Primary Raceme (Low) | 11, 24, 32, 33, 41, 54, 62, 72, 74, 78, 82, 84, 86, 87, 88, 89, 94, 96, 97, 101, 102, 117, 130, 135, 171, 182, 206, 208, 2012, 214, 220, 228, 235, 237, 239, 240, 241, 245, 246, 247, 248, 251, 252, 255, 256, 259, 262, 263, MIR, MPA |
| Flowering (Medium) | 62, 72, 78, 82, 89, 94, 96, 208, 240, 245, 251, 263, 265, SIP |
| Plant Architecture (Erect) | 13, 18, 25, 32, 33, 34, 44, 46, 52, 66, 69, 73, 74, 80, 81, 83, 87, 93, 94, 95, 117, 131, 132, 160, 165, 169, 170, 178, 181, 182, 195, 199, 204, 206, 211, 212, 219, 235, 236, 237, 240, 241, 244, 246, 248, 250, 251, 252, 253, 263, 265, MPA, SIP |
| Plant Stature (Very short) | 13,24, 36, 41, 56, 65, 78, 86, 102, 149, 159, 182, 183, 212, 239, 251, 262, 264, MPA |
| Fruit Dehiscence (Indehiscent) | 10, 31, 79, 81, 82, 84, 102, 122, 124, 134, 154, 185, 217, 246, 247, 257, 258, MIR |
| Number of Racemes Harvested (High) | 1, 2, 5, 7, 14, 17, 25, 31, 33, 38, 39, 40, 46, 48, 54, 55, 67, 72, 78, 79, 82, 89, 96, 97, 108, 112, 125, 126, 133, 134, 137, 138, 140, 141, 146, 148, 153, 171, 174, 177, 178, 180, 197, 199, 201, 202, 203, 209, 211, 216, 217, 220, 222, 223, 230, 231, 234, 235, 237, 238, 240, 242, 244, 245, 246, 247, 249, 256, 257, 263, 265, NOR, SIP |
| Seed Yield per Fruit (High) | 13, 25 |
| Weight of 100 seeds at 9 % moisture contente (High) | 3, 11, 31, 54, 69, 78, 79, 81, 82, 84, 86, 94, 97, 101, 135, 169, 170, 173, 179, 191, 231, 232, 237, 247, 256, 259, SIP |

Lines of castor bean from the germplasm bank of UFRB / CCAAB / NBIO, numbered from 1 to 265; and parental PAR (BRS 188 Paraguaçu), NOR (BRS 149 Northeast), MIR (Mirante 10), MPA (EBDA MPA-17) and SIP (Sipeal 28).
